# Mobile applications as a strategy to support parents in the care of newborns: a scoping review^*^

**DOI:** 10.1590/1980-220X-REEUSP-2022-0470en

**Published:** 2023-07-24

**Authors:** Juliane Pagliari Araujo, Adriana Martins Gallo, Cristina Maria Garcia de Lima Parada, Sonia Silva Marcon, Rosângela Aparecida Pimenta Ferrari, Keli Regiane Tomeleri da Fonseca Pinto, Adriana Valongo Zani

**Affiliations:** 1 Universidade Estadual de Londrina, Londrina, PR, Brazil.; 2 Universidade Estadual de Maringá, Maringá, PR, Brazil.; 3 Estadual Paulista Júlio de Mesquita Filho, Botucatu, SP, Brazil.

**Keywords:** Infant, Newborn, Mobile Applications, Parents, Access to Information, Smartphone, Recién Nacido, Aplicaciones Móviles, Padres, Acceso a la Información, Teléfono Inteligente, Recém-nascido, Aplicativos móveis, Pais, Acesso à informação, Smartphone

## Abstract

**Objective:**

To map and describe studies available in the literature about mobile applications to support parents in newborn care and data from applications accessible in online stores.

**Method:**

This is a scoping review following the Preferred Reporting Items for Systematic reviews and Meta-Analyses extension for Scoping Reviews guidelines. The searches were carried out in theses and dissertations databases and portals, in September 2021, and articles, theses, and dissertations were included. An independent search was performed in online stores of applications for operating systems A*ndroid* and *iOS*, in October and December 2021, and applications with content to support parents of newborns were selected.

**Results:**

A total of 5,238 studies and 757 applications were found, and of these, 16 and 150, respectively, composed the sample. The topics discussed in the studies were: care, breastfeeding, fever, identification of neonatal diseases, child growth and development. In the applications, the themes found were care, breastfeeding, growth, immunization, development, sleep, tips, and guidelines.

**Conclusion:**

Applications are important support tools for parents, as they are an innovative means and accessible to a large part of the population.

## RESUMO

**Objetivo:**

Mapear e descrever estudos disponíveis na literatura acerca dos aplicativos móveis para apoio aos pais no cuidado ao recém-nascido e dados de aplicativos acessíveis em lojas *online*.

**Método:**

trata-se de uma *scoping review* seguindo as orientações do Preferred Reporting Items for Systematic reviews and Meta-Analyses extension for Scoping Reviews. As buscas foram realizadas em bases de dados e portais de teses e dissertações, em setembro de 2021, e foram incluídos artigos, teses e dissertações. Realizou-se uma busca independente, nas lojas *online* de aplicativos nos sistemas operacionais *Android* e *iOS*, em outubro e dezembro de 2021, e selecionados aplicativos com conteúdo de apoio aos pais de recém-nascidos.

**Resultados:**

Foram identificados 5238 estudos e 757 aplicativos, e desses, compuseram a amostra 16 e 150, respectivamente. Os temas discutidos nos estudos foram: cuidados, amamentação, febre, identificação de doenças neonatais, crescimento e desenvolvimento infantil. Nos aplicativos, os temas encontrados foram: cuidados, amamentação, crescimento, imunização, desenvolvimento, sono, dicas e orientações.

**Conclusão:**

Os aplicativos são importantes ferramentas de apoio aos pais, pois são um meio inovador, além de estarem acessíveis a grande parte da população.

## RESUMEN

**Objetivo:**

Mapear y describir estudios disponibles en la literatura sobre aplicaciones móviles para apoyar a los padres en el cuidado del recién nacido y datos de aplicaciones accesibles en tiendas *online*.

**Método:**

es un *revisión de alcance* siguiendo las pautas de Preferred Reporting Items for Systematic reviews and Meta-Analyses extension for Scoping Reviewse. Las búsquedas se realizaron en bases de datos y portales de tesis y disertaciones, en septiembre de 2021, y se incluyeron artículos, tesis y disertaciones. Se realizó una búsqueda independiente en las tiendas *online* de aplicaciones en sistemas operativos *Androide e iOS*, en octubre y diciembre de 2021, y aplicaciones seleccionadas con contenido para apoyar a los padres de los recién nacidos.

**Resultados:**

Se identificaron 5238 estudios y 757 aplicaciones, y de estos, 16 y 150 conformaron la muestra, respectivamente. Los temas discutidos en los estudios fueron: cuidados, lactancia materna, fiebre, identificación de enfermedades neonatales, crecimiento y desarrollo infantil. En las aplicaciones, los temas encontrados fueron: cuidado, lactancia, crecimiento, inmunización, desarrollo, sueño, consejos y orientaciones.

**Conclusión:**

Las aplicaciones son herramientas de apoyo importantes para los padres, ya que son un medio innovador, además de ser accesibles para una gran parte de la población.

## INTRODUCTION

The use of mobile applications (Apps), among parents, to receive information and education about their children’s health, is more and more popular, and the use of computer technology is being increasingly accepted and used by society^(1)^. Main representative of the evolution of mobile telephony, the smartphone has as its main operating systems the *Android* and the *Iphone Operating System* (iOS)^(2)^.

Such a scenario collaborates for the construction of a new modality of health care, and the literature shows that Apps, including the information generated from them^(3)^, can be used to optimize results and reduce health risks in educational programs^(1,4),5^, as well as to understand the determinants that promote newborns’ (NB) health^(5)^.

Currently, the almost universal use of smartphones has presented a transformative potential for health care, as it allows users instant access to information through Apps, which have great potential to support health, facilitating access to information and communication between professionals and patients^(5,6)^.

Mobile health (mHealth) as a medical and public health practice supported by mobile devices^(7)^ has been gaining prominence, due to the possibility of interventions and behavior change, through health promotion, self-management of diseases or conditions, data monitoring, and provision of information and communication^(6,7)^.

App-based interventions available for smartphones have become an increasingly valuable resource for disease prevention^(8)^. As an ally, mobile technology should be used for training users, as a source of information for parents, and for continuity of care, considering the large number of births, including premature births, and the importance of providing health care and home care^(9)^.

Nursing is present in the care of newborns and their families at all levels of care and the experience of these professionals in the development of mobile Apps contributes to safe care and care practice^(10)^. Thus, knowledge of the already available Apps to support parents of newborns is required, as well as the studies conducted in this field, to encourage professional practice and guide parents on this topic.

In this context, the growing number of smartphone Apps available^(10)^ and the increasing inclusion of health professionals in the construction and validation is apparent. There is a variety of purposes in the development and use of the App and health researchers have focused on this theme, bringing to light its benefits, presenting results such as theses and dissertations linked to graduate programs in nursing in Brazil^(10)^ and in the world.

Aiming at contributing to the consolidation of knowledge regarding the development and availability of mobile Apps as a support strategy for parents of newborns, the objective of this research was to map and describe available studies in the literature about mobile applications to support parents in caring for the newborn and app data accessible in online stores.

## METHOD

### Design of Study

This is a scoping review whose protocol was published^(11)^ and registered in *Open Science Framework* (OSF), available at https://osf.io/vtyce/, and conducted with methodological rigor, recommended by the *Joanna Briggs Institute* (JBI)^(12)^, following the recommendations of the *Preferred Reporting Items for Systematic reviews and Meta-Analyses extension for Scoping Reviews* (PRISMA-ScR) Checklist^(13)^. Scoping reviews are not intended to assess the quality of available evidence but aim to map the main concepts that support a research area, involving a systematic procedure^(14)^.

### Data Collection

The structure of this review took place in two phases, the first being a search in databases and the second applied in online mobile devices stores. The first phase was subdivided into six stages: 1) identification of the question and study objective; 2) identification of relevant studies that would allow the extent and comprehensiveness of the review’s purposes; 3) selection of studies, according to predefined criteria; 4) data mapping; 5) summarization of results, through a qualitative analysis in relation to the objective and question; 6) presentation of results. For the second phase, an independent search was carried out in the mobile App virtual stores, through smartphones, with operating system *Android and iOS,* to identify the Apps that addressed the theme to support NB parents.

The elaboration of the guiding question was based on the mnemonic structure PCC (Population, Concept and Context) proposed by the JBI, with Population (P) being newborn, parents and family; Concept (C), studies addressing mobile applications in the field of neonatology; Context (C), care for newborns. Following this organization, the research question was elaborated: Which mobile applications, developed to support parents in newborn care, exist in the literature or are available in online smartphone stores with the operating systems *Android* and *iOS*?

To ensure the reliability of the process, the searches and selection of scientific productions and applications were carried out by two independent investigators, who standardized the sequence of procedures and, after completing the sample recruitment, compared their findings to check for discrepancies in the sample obtained. A third investigator was called in cases of non-agreement.

Studies available in an electronic publication in the databases *PubMed, Cumulative Index to Nursing and Allied Health Literature (CINAHL), Web of Science, Scopus*, Latin American and Caribbean Literature on Health Sciences (LILACS), *Excerpta Medica dataBASE (Embase), Cochrane Library,* Google Scholar, *Scielo*, Portal of the Virtual Health Library (VHL) were used, as well as those in the gray literature (dissertations and theses), through the Portal of Theses and Dissertations from Latin America and the Catalog of Theses and Dissertations of the Coordination for the Improvement of Higher Education Personnel (CAPES).

The databases were searched through the CAPES plat­form on the Federated Academic Community (CAFe) portal on September 16, 2021, using the “advanced search” feature. Controlled and uncontrolled descriptors in Health Sciences (DeCS), *Medical Subject Headings (MeSH), and CINAHL Headings*, using the Boolean operators “*AND*” and “*OR*” were consulted.

**Chart 1 –:**
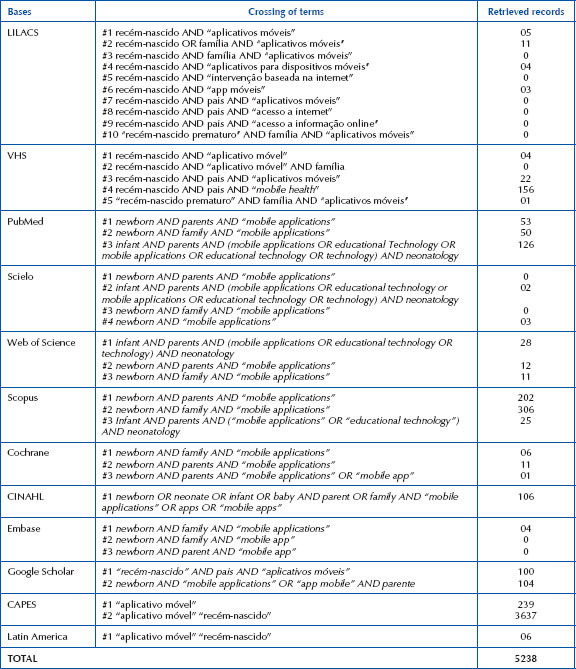
Development of search strategies in databases. Londrina, PR, Brazil, 2023.

Source: Prepared by the authors.

The strategy integrated descriptors crossed with each other in Portuguese and English, as well as uncontrolled descriptors (Chart 1).

### Eligibility Criteria

Original scientific articles and reviews, either descriptive or analytical, quantitative, or qualitative, theses and dissertations electronically available in full, which dealt with the theme and without time or language limitations, were chosen. Findings addressing NB, premature infants and children, parents and professionals were also included, as well as studies bringing other support tools besides the App. Scientific productions in editorial format, letter to the editor, opinion articles and advertisements were excluded; gamified apps, for the exclusive use of professionals, with an exclusive child theme, and duplicate documents were computed only once.

The fourth stage consisted of reading the title and abstract, to identify whether they answered the research question. After this reading, the pre-selected studies were read in full to confirm inclusion in the final sample and made up the fifth stage, which consisted of completing and evaluating the data collection instrument. The instrument was based on the JBI^(12)^ requirements and adapted for the present review, comprising: author, year, methodological design, main results, parent support theme and usability area, and description of the App’s content.

In the second phase, independently of the search in the databases, searches were carried out in online mobile app stores for smartphones with operating systems *Android*, on October 28, and *iOS*, on December 26, 2021, according to the protocol^(11)^. Thus, the Apps available in those stores were also the results of this review, which had as an inclusion criterion addressing support content for parents of newborns. The apps covering newborns, infants, and information for parents and professionals were also included. The exclusion criteria adopted were: not being available for download in free or paid forms, gamified App, unrelated to the theme, exclusive for professionals and product sales. The Apps found twice, during the search with different terms and stores, were counted only once.

In each virtual store, the searches took place using the term “newborn”. Then, a second search was performed with the term “premature” individually. This choice was due to the specificity of meeting the search criteria in online stores. All Apps found were downloaded, 485 of which being found in the operating system *Android* and 272 in the system *iOS*. This stage of the research was also carried out by two independent investigators, simultaneously, to avoid selection bias. A total of 150 Apps with themes related to support to newborns’ parents were chosen.

For data collection, an instrument consisting of ten variables about the App was used: operating system; country/state in which it was produced; language; year of update in the virtual store; parent support theme; indicative rating of the content; entity licensed to use the application; type of access (open or paid); accessibility for people with disabilities; number of downloads. It should be noted that such variables were used because they are information about the App that can be obtained and are available in the App itself and/or in the online store.

### Data Extraction and Analysis

Then, in the sixth stage, the data from the studies and applications were interpreted separately, with the studies being compared and based on theoretical knowledge and data extracted from mobile applications entered a spreadsheet and then exported to the R program, version x64 4.0.0. For this analysis, descriptive statistics were used, expressed by absolute and relative frequencies.

### Ethical Aspects

There was no need for ethical appreciation, as the study analyzed secondary and already publicly available data. It should be noted that the copyright of the cited studies was respected.

## RESULTS

**Figure 1 –:**
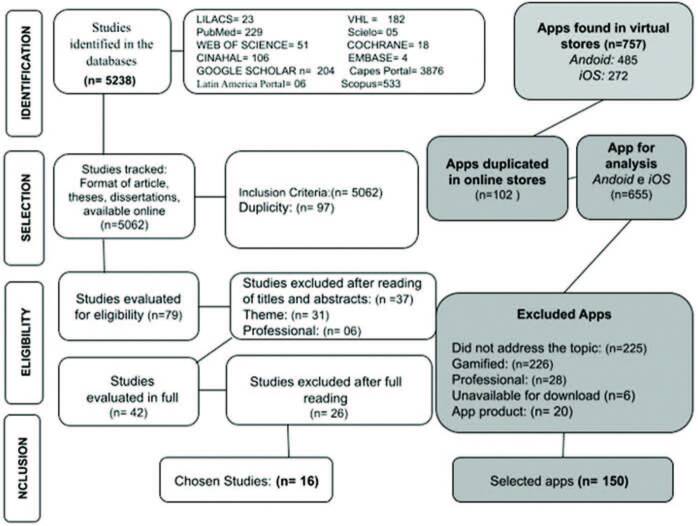
Flowchart of the selection process of studies in databases and applications in virtual stores. Londrina, PR, Brazil, 2023.

For the description of the selection process, the flowchart *Preferred Reporting Items for Systematic Review and Meta-Analyses* (Prism) was used, which was adapted for this study (Figure 1). The database search mapped 5,238 potentially eligible studies, with 16 remaining in the final sample. Among the apps available in online stores, 758 were identified, of which 150 were selected.

The narrative synthesis of the 16 studies selected points to the main theme addressed: support for the parents of NBs, care of NBs^(15–21)^, breastfeeding (BF)^(22–25)^, fever^(26,27)^, child development^(28)^, growth^(29)^, and identification of neonatal diseases^(30)^. In addition, 11 scientific productions were presented in the form of articles and 5 dissertations and were published between 2017 and 2021.

**Chart 2 –:**
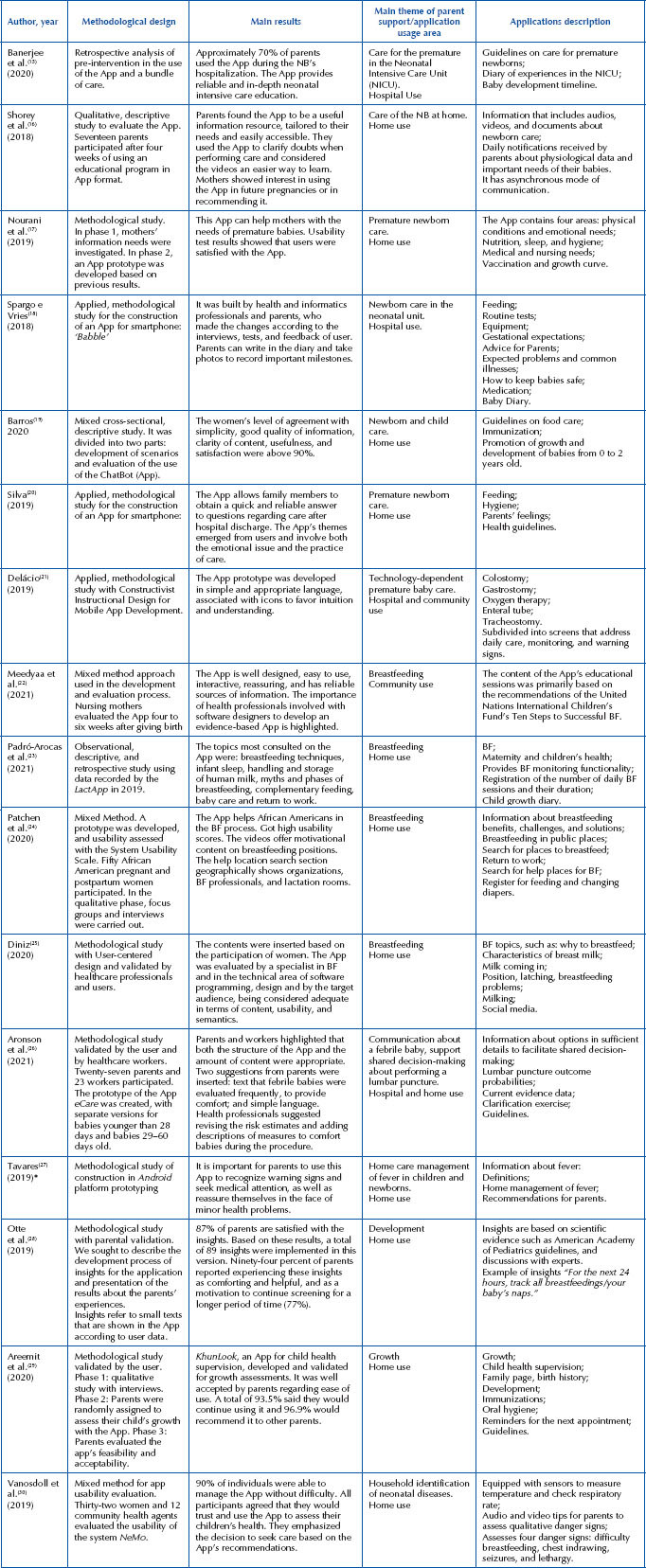
Results of selected studies. Londrina, PR, Brazil, 2023.

Source: Prepared by the authors *includes NB in the study.

The studies were produced in Brazil^(19-21,25,27)^, United States of America^(24.26)^, United Kingdom^(15)^, Netherlands^(28)^, Singapore^(16)^, Australia^(22)^, Thailand^(29)^, Iran^(17)^, Uganda^(30)^, New Zealand^(18)^, and Spain^(23)^ (Chart 2).

**Chart 3 –:**
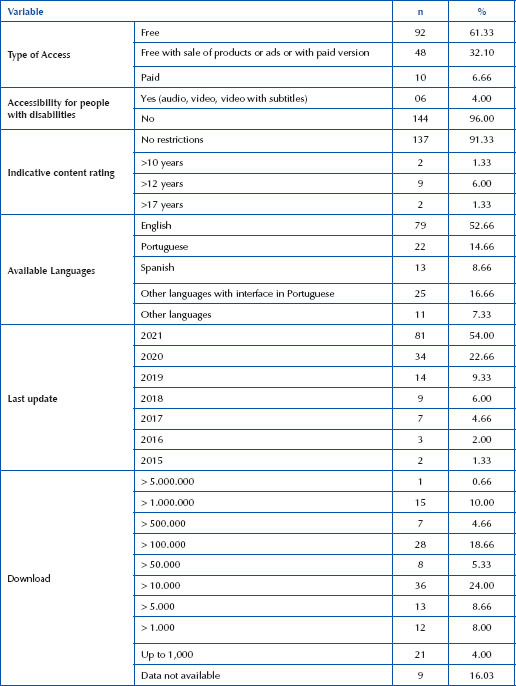
Characterization of mobile applications available in online stores, to support parents of newborns. Londrina, PR, Brazil, 2023.

Source: Prepared by the authors.

The search in virtual stores resulted in a sample of 150 applications, available for smartphones, and the characterization of these App is found in Chart 3.

**Figure 2 –:**
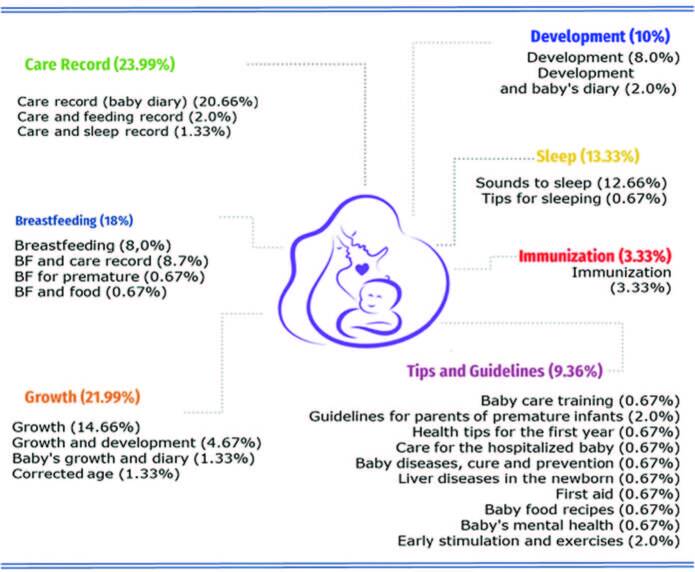
Apps Newborn Parent Support Themes. Londrina, PR, Brazil, 2023.

Figure 2 shows the topics addressed in the Apps selected to compose the study sample.

## DISCUSSION

With the studies selected in this scoping review, it was possible to systematize, detail, and highlight central aspects related to the mobile Apps to support the parents of NB, advancing in discussions, in an attempt to point out an overview of this universe that allows the structuring of knowledge and the practice of nursing in this context.

It should be noted that, in neonatology, childcare, in a way, generates uncertainty^(31)^. Thus, mobile technology adds to the support for parents of newborns. Apps are designed to be performative, that is, they encourage and provoke users to act, for example, to change certain health-related behaviors^(28)^. Sixteen studies discussing the theme of support for parents of newborns and 150 Apps (after search in virtual stores), were mapped, described, and elected. Of these, 91.33% are rated as containing no restrictions for use, 31.32% are available and can be accessed in Portuguese, 54% had the last update in the year 2021, 10.66% have more than 1,000,000 downloads, and only 6.66% have charged content access.

Among the themes identified, care for the NB in different contexts, involving hospital^(15,18),21^ and domestic care^(16,17,19–21)^, and breastfeeding^(22,23),25^ are highlighted, findings that converge with Apps available in online stores (23.99% and 18% respectively). Most of the Apps analyzed, related to baby care, refer to daily care, for personal records and tasks such as bathing, changing diapers, feeding, among others. Fever^(26.27)^, child development^(28)^, growth^(29)^, and identification of neonatal diseases^(30)^ were also support themes identified in the studies.

Information about daily care is a useful tool for the parents’ effective participation^(32)^ and quality care in the family context is essential for the NB’s healthy growth and development^(33)^. Scientific evidence indicates that preventive practices can significantly reduce neonatal morbidity and mortality, including basic care such as providing warmth, hygiene, and exclusive breastfeeding^(33)^.

Many parents say the App facilitates the quick and reliable retrieval of essential information^(16)^, being useful to optimize knowledge^(17)^ and take care of their child after hospital discharge^(16,17),20^. During the NB’s hospitalization, the parents also state that the use of the App, together with a bundle of care, helps to gain knowledge and significant confidence to enable them to care for their babies in the NICU and, consequently, antedate hospital discharge^(15)^.

The literature shows that some Apps are also being developed to support parents of preterm infants, who need specific care after hospital discharge, ranging from App guidance on care^(17–21)^ to greater interaction, such as monitoring growth and development^(9)^ and identifying neonatal diseases remotely^(30)^, care with nutrition, sleep and hygiene, vaccination and growth curve^(17)^. Parental participation in care, guided by the use of an App, based on scientific evidence, can help reduce the hospitalization rate and improve the quality of care^(17)^.

In their turn, BF apps provide specific instructions on breastfeeding, as well as important information involving the family and self-management tools for breastfeeding mothers^(7,22,23,25,34,35)^.

The main factors indicative of initial difficulties with the breastfeeding technique are inadequate latching (25.0%), the baby’s response to contact with the breast (26.1%), and problems with the breast (28.3%)^(36)^, and 92% of mothers had complications in breastfeeding, including difficulty, pain, and concern about the amount of milk^(7)^. Thus, information and support for breastfeeding mothers is vital to encourage breastfeeding.

The practice of breastfeeding can also be influenced by the participation and knowledge of fathers, and the greater the knowledge about the benefits of breastfeeding, associated with support and involvement, the better the practice of women who provide breast milk to their children, as a well-informed father/mother becomes a key element in the maintenance and success of breastfeeding^(37,38)^.

Apps related to breastfeeding, in general, aim to provide support information to the woman and allow the creation of a profile with a diary of personal records^(25)^ about time, duration of feeding, and a reminder of breastfeeding time^(23,24)^, observations about child behavior and baby care^(23)^, location of milk collection points, lactation rooms, and public spaces favorable to breastfeeding^(24–35)^. Advances in the testing of Apps are still required, in what regards breastfeeding, especially related to content, acceptability, effectiveness, usability, clarity regarding standardization in operational development, validation by women, and all these based on scientific evidence^(25,34)^.

The mobile Apps that offer support to parents regarding care in case of fever^(26,27)^ and identification of neonatal diseases^(30)^ were developed to provide simple language, focusing on the integration of parents in care and decision-making^(26,27)^, highlighting the importance of Patient- and Family-Centered Care^(26–28,30)^. It is known that developing a support and information App for parents, with themes that can cause stress and without exacerbating their fear or anxiety^(26)^ is a challenge.

The audiovisual resources in the App are important tools^(24,30)^; however, in the App available in online stores, selected in this study, only 4% had these resources. Although some health professionals have suggested making videos of NB procedures available, many parents have stated that watching such material would be disturbing or alarming^(26)^. It should be noted that some Apps allow parents to interact with health professionals and this leads to a sense of safety in the postnatal period and brings greater satisfaction to users^(15,16),30^.

The Apps that were built by health professionals, based on scientific evidence,^(19,22–25)^ with the participation of users^(28)^ and validated by them, had good acceptance of use^(9,16–18,22,24,26,30)^.

The studies focus on the feasibility and acceptability of mobile Apps, although it is necessary to advance in the evaluation of their effectiveness^(34)^. In addition to this discussion, it is necessary to evaluate Apps as a support tool for parents that can be used in the long term, since monitoring the child’s growth and development is an important indicator of quality of life and child health. Therefore, in Apps with an area for home use, those related to child growth^(29)^ (10%) and development^(28)^ are highlighted (21.99%).

Based on the mapped evidence, it is possible to state that health professionals are increasingly engaged in the development of health-related Apps and, considering the number of downloads, parents are increasingly accessing this technology. Sharing this health information strengthens the practice of ­evidence-based care for newborns.

The scoping review method was adopted for allowing the selection of different types of studies, enriching the findings and highlighting the applications developed to support the parents of newborns. It is clarified that the review has limitations regarding searches in online stores. Therefore, there are App differences from a smartphone to another, in what regards the operating system and the update of these mobile devices.

## CONCLUSION

The present study allowed mapping and describing the mobile Apps available to support NB parents. These are important support tools, as they are an innovative means and a method capable of generating interest, besides being accessible to most of the population, due to the widespread use of mobile devices connected to the Internet.

Knowledge of the technology of Apps available to parents of NBs by nursing professionals can significantly contribute to engagement and guidance on the use of these Apps in NB care, expanding the quality of nursing care in the maternal and child area.

It should also be highlighted that it is important and necessary that new Apps developed by health professionals are based on scientific evidence and validated by peers in what regards content, semantics, functionality and usability, and viewing their potential to be integrated into professional practice, even allowing optimization of communication between nurses and parents of newborns from the perspective of care.
